# Loudness affects motion: asymmetric volume of auditory feedback results in asymmetric gait in healthy young adults

**DOI:** 10.1186/s12891-022-05503-6

**Published:** 2022-06-17

**Authors:** Julia Reh, Gerd Schmitz, Tong-Hun Hwang, Alfred O. Effenberg

**Affiliations:** Institute of Sports Science, Leibniz University Hannover, Am Moritzwinkel 6, 30167 Hannover, Germany

**Keywords:** Sonification, Asymmetric volume, Gait pattern, Auditory feedback, Ground contact time

## Abstract

**Background:**

The potential of auditory feedback for motor learning in the rehabilitation of various diseases has become apparent in recent years. However, since the volume of auditory feedback has played a minor role so far and its influence has hardly been considered, we investigate the volume effect of auditory feedback on gait pattern and gait direction and its interaction with pitch.

**Methods:**

Thirty-two healthy young participants were randomly divided into two groups: Group 1 (*n* = 16) received a high pitch (150-250 Hz) auditory feedback; group 2 (*n* = 16) received a lower pitch (95-112 Hz) auditory feedback. The feedback consisted of a real-time sonification of the right and left foot ground contact. After an initial condition (no auditory feedback and full vision), both groups realized a 30-minute habituation period followed by a 30-minute asymmetry period. At any condition, the participants were asked to walk blindfolded and with auditory feedback towards a target at 15 m distance and were stopped 5 m before the target. Three different volume conditions were applied in random order during the habituation period: loud, normal, and quiet. In the subsequent asymmetry period, the three volume conditions baseline, right quiet and left quiet were applied in random order.

**Results:**

In the habituation phase, the step width from the loud to the quiet condition showed a significant interaction of volume*pitch with a decrease at high pitch (group 1) and an increase at lower pitch (group 2) (group 1: loud 1.02 ± 0.310, quiet 0.98 ± 0.301; group 2: loud 0.95 ± 0.229, quiet 1.11 ± 0.298). In the asymmetry period, a significantly increased ground contact time on the side with reduced volume could be found (right quiet: left foot 0.988 ± 0.033, right foot 1.003 ± 0.040, left quiet: left foot 1.004 ± 0.036, right foot 1.002 ± 0.033).

**Conclusions:**

Our results suggest that modifying the volume of auditory feedback can be an effective way to improve gait symmetry. This could facilitate gait therapy and rehabilitation of hemiparetic and arthroplasty patients, in particular if gait improvement based on verbal corrections and conscious motor control is limited.

## Background

The ability to perceive noise and sound is of great importance for our everyday interaction with the environment. For example, auditory perception helps us to recognize and determine distances, speeds, obstacles, materials, and our own position in space [1–4]. In sports, acoustic signals, sounds, verbal agreements, and music are often used to synchronize and modulate movements. Sounds are produced by movement, e.g. when bouncing off spring floors, hitting balls and when arms and legs hit water, or are consciously generated, e.g. when the starting shot is given or when shouting in team sports. The volume of sounds is often causally related to the intensity of movement. Thus, greater energy means increased power and acceleration or deceleration resp., which results in increased volume.

Possibly due to these physical correlation between movement and sound, neurophysiological findings suggest a close relationship between the movement system and auditory brain areas. Several imaging studies have shown that noises or sounds produced by a known movement induce neuronal activation in the human brain that resembles the neuronal activation during execution of the action. This simulation can be observed especially in the mirror neuron system and has become known in recent years under the term “action-listening” [5–8]. Furthermore, Chen et al. [9] showed in two fMRI experiments that rhythmic sounds generally cause an activation of the motor cortex in humans. The participants of experiment 1 knew the task of tapping on a right mouse button in synchrony to different rhythms given by a computer and via headphones from an exercise session on the day before the fMRI measurements. In contrast, participants of experiment 2 did not know that they were supposed to tap to the rhythms during the course of the fMRI measurement. Since no practice session was conducted on the previous day, they only learned about the tapping task after they had passively listened to the rhythms once. Under both conditions, listen with action anticipation and passive listening, the supplementary motor area, mid-premotor cortex (mid-PMC), and the cerebellum were activated.

It also became clear that people are better able to recognize the sound pattern generated by their own actions than a sound pattern generated by other persons actions and to assign it to themselves [10–13]. For auditory perception, therefore, a close perception-action link can be assumed in humans. Due to the intrinsic connection between sound and movement in space and time [14–17] and the neural connectivity described above, it seems reasonable to use auditory information to provide targeted and effective feedback for sports training and motor (re-)learning.

In the research on motor behavior, there exist many different approaches regarding the artificial generation of augmented auditory feedback (AAF). The following AAF methods were mainly considered: natural movement sounds [18–20], error feedback [21–23], rhythmic auditory stimulation [24–27], sonification [28–34] and musical movement feedback [35–37]. It has been shown that AAF is effective in a wide variety of application areas. There is evidence of efficacy in sports, e.g. rowing [38, 39], skiing [40], golf [41], cycling [42], and swimming [43], and also in movement rehabilitation, particularly in Parkinson’s disease [44–46] and stroke patients [47, 48].

So far, the choice of one of the aforementioned AAF methods and the mapping of acoustic parameters to specific movements, seems to be based primarily on the assessment of the movement or disease under investigation. For example, for gait rehabilitation in Parkinson’s patients [49], rhythmic-auditory stimulation was investigated above all, since walking is an intrinsically rhythmic and repetitive movement. For movements with more degrees of freedom, such as attack-and-release actions (e.g. grasping), studies were conducted more frequently using real-time movement sonification or musical sonification [43, 45].

Movement sonification (MS) means the transformation of kinematic human motion data into sound, resulting in multidimensional motion acoustics. So far, research on gait sonification mainly considered timbre and pitch [50–53], rhythm [54–58] and tempo [59, 60]. As far as known, even if correlations of volume and distance [61], volume and size of objects [2, 62, 63], volume and direction and speed of movement [64, 65] as well as volume and articulatory kinematics [66] are known from other research areas, these have hardly been included when using gait sonification. However, due to the known correlations, volume could be an easy-to-use parameter, for example, to specifically treat rehabilitation patients with asymmetrical gait (stroke patients, unilateral arthroplasty) with the help of well-shaped auditory feedback.

In a recent review paper, Schaffert et al. [67] point out that the question of “what auditory components and amount of information are most relevant for motor training and rehabilitation” has not yet been sufficiently investigated. Among other things, it is unclear what effect individual parameters of sound (e.g., pitch, volume, timbre, tempo, rhythm) have on the execution of movement and motor control (cf. also [68]). However, knowledge of the concrete impact of the various sound parameters in AAF considering different target groups would make the use of auditory feedback more purposeful and efficient in the future. This work aims to contribute to the clarification of the sound-parameter-motion relationship in AAF. For this purpose, we consider the parameters volume and pitch and their possible influence on the gait pattern of healthy young persons. These two parameters are taken into account since pitch and loudness perception are correlated due to the perceptual range of the human auditory system: We hear sounds loudest at frequencies between 2000 and 4000 Hz, and sounds below or above are perceived more quietly at the same sound pressure level [69]. Furthermore, correlations between pitch and range and direction of motion [16, 70–73] are well known and clearly described in the literature. A higher pitch is usually accompanied by an increase in height and velocity which also indicates a similarity to volume perception.

This study intends to investigate the influence of different volume and its interaction with pitch of real-time sonification of the ground contact on the gait pattern of healthy persons.

First, the overall volume was varied by 6 dB in three steps (loud 0 dB, normal − 6 dB, quiet − 12 dB) to determine its influence on participants’ gait pattern (stride width, stride length, gait speed). Second, we hypothesized that the asymmetric loudness of sonification influences the gait symmetry of the participants. In this regard, the volume difference was varied between the right and left channel of the headphone used. Furthermore, to investigate whether pitch interacts with volume, the volume changes were applied to two groups (G1 *n* = 16, G2 *n *= 16) with different sonification pitches: G1 received a sound with a base frequency of 150-250 Hz and G2 received a sound with a base frequency of 95-112 Hz.

## Methods

### Participants

A total of 32 young, healthy volunteers participated in the study. Each participant was informed about the general course of the study and the handling of the data collected before the start of the measurement. Written informed consent was obtained from each participant. The study was conducted in accordance with the guidelines stated in the Declaration of Helsinki and the regulations of the Ethical Committee of the Leibniz University Hannover (EV LUH 15/2019). Volunteers aged 18-35 years with normal physiological walking and hearing ability were included in the study. Acute injuries or pain of the lower extremities and diseases affecting hearing, vision or balance were defined as exclusion criteria. The criteria were checked by means of a questionnaire, which was completed by the participants before the start of the measurements. In addition, each participant obtained a hearing test (HTTS hearing test software, Version 2.10, SAX GmbH, Berlin, Germany) to ensure sufficient hearing ability and well-balanced hearing in both ears.

Participants were randomly divided into two groups. G1 (*n* = 16, gender: 8 m/8f, age: 23.6 ± 3.4 years, height: 178.3 ± 9.7 cm, weight: 71.3 ± 15.6 kg, weekly sport activity: 6.4 ± 3.8 h) received a high pitch sonification and G2 (*n* = 16, gender: 9 m/7f, age: 25.2 ± 3.3 years, height: 180.1 ± 7.1 cm, weight: 73.3 ± 10.0 kg, weekly sport activity: 6.2 ± 2.9 h) received a lower pitch sonification. T-tests for independent samples of the baseline characteristics of both groups revealed no significant differences between G1 and G2 (age *p* = 0.202, height *p* = 0.552, weight *p* = 0.669, weekly sport activity *p* = 0.836). The proportion of right- and left-handed and right- and left-footed participants was approximately balanced in G1 and G2 (G1: 14 right-handed, 2 left-handed; 7 support leg right, 9 support leg left; G2: 13 right-handed, 2 left-handed, 1 ambidextrous; 5 support leg right, 11 support leg left).

In order to capture whether there are different emotional responses in participants due to the different pitch of sonification, the Bf-SR questionnaire was used to assess mental state [validated German questionnaire Bf-SR [74]]. The questionnaire was filled out by the participants once before the start of the gait measurements and once after the gait measurements.

### Experimental design

The measurements took place in a quiet gym of the Leibniz University Hannover. Each participant participated in one 90-minute measuring session. A randomized single-blinded design was chosen. Unlike the supervisor of the experiment, the participants were not informed in advance about their group allocation and the different volume conditions. Each participant went through all of the conditions presented below in random order.

**Fig. 1:**
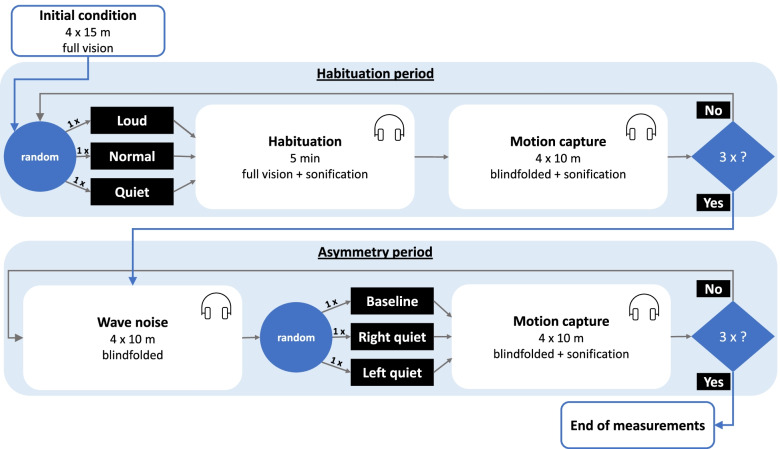
Experimental design. The experiment starts with an initial condition, followed by the habituation period (top) and the asymmetry period (bottom), each consisting of three repetitions (blue diamond). The three repetitions include three different volume conditions in the habituation period (loud, normal, and quiet) and in the asymmetry period (baseline, right quiet, left quiet), each run once in randomized order

The measurements began with an initial condition: the participants walked four times straight from a start mark towards a target at a distance of 15 m with full vision and without sonification. The further course of the experiment was divided into two periods: a habituation period and an asymmetry period. In both periods the participants received sonification via headphones while walking. The sonification of the right ground contact was played only on the right speaker of the headphone and the sonification of the left ground contact on the left speaker of the headphone. In detail, the sonification mappings are described in section *Ground contact sonification*. Both periods consisted of three blocks each. During the habituation period, the volume was varied symmetrically on both sides: (1) loud, (2) normal, (3) quiet. During the asymmetry period, the volume was varied asymmetrically: (1) right quiet (RQ), (2) left quiet (LQ), (3) right and left equal (baseline). In the habituation period, one block consisted of a five-minute walking phase in which the participants walked back and forth between start and target with full vision and sonification (loud, normal, quiet). This was followed by four times walking blindfolded from the start towards the target under the same volume condition as during the five-minute gait phase. In the asymmetry period, one block consisted of four blindfolded walks from the start to the target with wave noise. This was followed by four blindfolded walks from the start to the target with sonification (RQ, LQ, baseline). The course of the experiment is shown in Fig. 1.

### Gait analysis

**Fig. 2:**
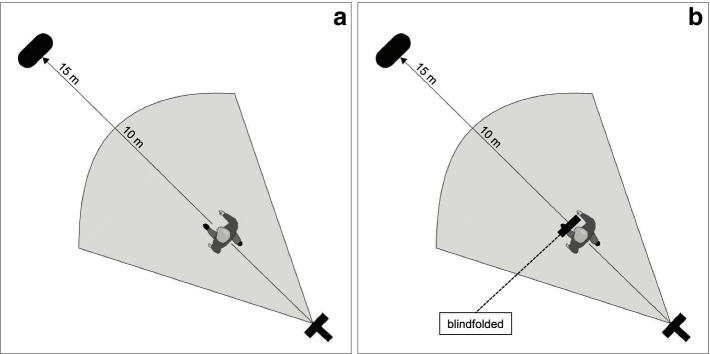
**a** Experimental setup for the gait measurements. The start-calibration mark is on the bottom right. At the top left is the target marking and the 10 m distance is marked by an arc line. **b** In the initial condition and habituation, participants walk with full vision. Right side: In the conditions loud, quiet, normal, baseline setting, RQ setting, LQ setting, and wave noise, participants walk blindfolded

To ensure consistent walking conditions, the test persons were provided with anti-slip socks in which they could walk safely in the gym. A start marker was attached to the floor and a red target point was attached to a box (70 × 50 × 40 cm) to clearly delimit the walking area (Fig. 2). The markings indicated a distance of 15 m. Furthermore, a white line drawn in an arc on the ground marked a distance of 10 m from the starting point. The participants were fitted with the wireless motion analysis system MVN Awinda (XSens Technologies B.V., Enschede, the Netherlands). Seven inertial measurement units (IMUs) were attached to the sacrum (1 IMU), lateral side of both femurs (2 IMUs), medial surface of tibias (2 IMUs), and middle arches of the feet (2 IMUs) using velcro straps. The data acquisition was carried out using the software MVN Studio BIOMECH (Version 4.1, XSens Technologies B.V., Enschede, the Netherlands), which stores the data at a frequency of 60 Hz. Before each gait recording, the motion analysis system was calibrated directly at the marked starting point to ensure the highest possible measurement and sonification accuracy.

The measurements started with an initial condition without visual restriction and without sonification. The participants approached the target four times at a self-selected average speed and stopped about 5 cm before the target mark. The kinematics of the total distance of 15 m was recorded. This was followed by the habituation period. For the five-minute walking phases without visual restriction the participants were instructed to put on the wireless headphones after calibration and to walk back and forth between the start and finish markings for 5 min each.

All other conditions (loud, normal, quiet, wave noise, baseline, RQ and LQ) were performed blindfolded. Before each condition, the participants were instructed to first concentrate visually on the target point, second to put on headphones, third to put on the sleeping mask and fourth to start walking within 5 s. In all blindfolded conditions, the participants were stopped at a 10 m line by a touch on their back to achieve a standardized walking distance. Data acquisition was also stopped at this point. The headphones were removed from the participants heads, but not the sleeping mask, in order to avoid the possibility of conscious directional correction during subsequent attempts. The participant was guided back to the starting point via the touch on the back, where the sleeping mask could be taken off again.

### Ground contact sonification

**Fig. 3:**
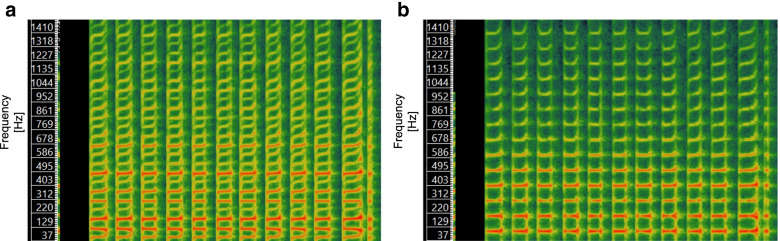
**a** Melodic range spectrogram of the sound used for the sonification of the ground contact for G1 (base frequency of 150-250 Hz) and **(b)** melodic range spectrogram of the sound used for the sonification of the ground contact for G2 (base frequency of 95-112 Hz). Only one channel is shown at a time. The spectrograms were generated using Sonic Visualiser (Release 4.3, Centre for Digital Music at Queen Mary, London, GB)

For sonification, the kinematic data was streamed in real time from the MVN Biomech software to a self-developed Spyder program (Version 3.3.1., The Scientific Python Development Environment, Spyder Developer Community). Latency from touch down to sound occurrence was less than 100 ms. An algorithm was used to determine the gait events touch-down (TD) and toe-off (TO) using the acceleration data of the feet. The sonification of the ground contact time (from TD to TO) was performed by an implemented CSound module (Csound 6, LGPL). One channel was used for each foot, so that on the left ear only the ground contacts of the left foot and on the right ear only the ground contacts of the right foot could be heard. The pitch was the same on both sides. G1 received sonification of ground contact times with a base frequency of 150-250 Hz. The sound resembles the noise produced when walking through snow. However, the sound has more characteristics of a tone. G2 received sonification of ground contact time with a base frequency of 95-112 Hz. Due to the narrower frequency setting, the sound appeared deeper and softer, and its frequency spectrum was more clearly delineated from the first one. Both sounds are visually contrasted in a Melodic Range Spectrogram in Fig. 3.

The loud volume level (59.0 dB) was determined by pilot measurements in which five young healthy participants were asked whether they perceived the sound clearly when walking 10 m. If the sound was perceived as too loud, they were immediately stopped, and the volume was reduced gradually via the headphones. Steps of volume decrease was chosen such that differences were not clearly noticeable to avoid participants responding with a deliberate change in gait pattern. After the measurements, participants were asked whether they perceived differences in gait sonification, which was the case for only three of the 32 participants (G1: 1; G2: 2).

The volume change of the ground contact sonification was implemented by a decibel change in CSound. This change was based on the inverse square law, which according to Blauert [61] states that the sound pressure level decreases by approximately 6 dB when the distance is doubled. The loud setting was defined in CSound as 0/0 dB (sonification 1/1 = 100%), the normal setting as − 6/− 6 dB (sonification 0/0 = 50%) and the quiet setting as − 12/− 12 dB (sonification − 1/− 1 = 25%). Accordingly, the RQ setting was defined as − 12/− 6 dB and the LQ setting as − 6/− 12 dB. This resulted in actual mean sound pressure levels of 52.0 dB (quiet), 55.5 dB (normal), and 59.0 dB (loud). The volume settings of the headphones and the laptop used were kept the same throughout the experiment.

### Data processing

Six middle steps of each gait recording were cut in MVN Studio BIOMECH and included in the evaluation in order to exclude any falsification by accelerating and stopping at the beginning or end of the walk. The gait events TD and TO were determined using a self-developed algorithm in MATLAB (R2016a, The MathWork inc., Natick, MA, USA) and the gait parameters stride duration, percentage step duration in relation to stride duration, percentage ground contact time in relation to stride duration, stride speed, cadence, stride length, step length and step width were analyzed. We defined one stride as the range between the TD of 1 ft to the following TD of the same foot. One step was defined as the range from the TD of 1 ft to the following TD of the other foot and the ground contact time was the time between TD and TO of the same foot. The percentage step duration and the percentage ground contact time were considered in relation to the stride duration, i.e. the stride duration was defined as 100%. The step width is the distance between both feet orthogonal to the direction of gait and the cadence is defined as number of steps per minute.

For the evaluation of the gait direction the recordings were not cut. The target position is the position of the participants’ feet in the initial condition, which was measured for each participant at the beginning. The stop position is the final foot position of the participants in the habituation period and asymmetry period, when walking blindfolded. The direction of gait was determined in MATLAB by establishing a line equation based on the start position and target position of the feet (Eq. 1). The amount of the angle between the two vectors target position and stop position was determined by Eq. 2.

1 $$\Delta y={y}_{stop}-\left(\ \frac{y_{target}-{y}_{start}}{x_{target}-{x}_{start}}\bullet \left({x}_{stop}-{x}_{start}\right)+{y}_{start}\right)$$

2 $${\alpha}_{dev}={\cos}^{-1}\frac{\left({\overrightarrow{\Delta s}}^{{}^{\circ}}\ \overrightarrow{\Delta t}\right)}{\left|\overrightarrow{\Delta s}\right|\bullet \left|\overrightarrow{\Delta t}\right|}$$

In Eq. 1, ∆*y* is the difference between the y-coordinates of the stop vector and target vector at the same level, *y*_*stop*_ is the y-coordinate of the stop vector, *x*_*stop*_ is the x-coordinate of the stop vector, *y*_*target*_ is the y-coordinate of the target vector, and *x*_*target*_ is the x-coordinate of the target vector. A ∆*y* > 0^°^ was defined as a deviation to the left, a ∆*y* < 0^°^ was defined as a directional deviation to the right.

In Eq. 2, *α*_*dev*_ is the amount of the directional deviation, $\overrightarrow{\Delta s}$ is the stop vector, and $\overrightarrow{\Delta t}$ is the target vector.

To determine the ratio the data of the conditions RQ and LQ were each divided by the baseline condition (asymmetry period) and the data of the conditions loud and quiet were each divided by the normal condition (habituation period). For the statistical analysis, the ratio of stride duration, step duration, ground contact time, percentage step duration, percentage ground contact time, stride speed, cadence, stride length, step length and step width were considered. For the gait direction, the angles of the conditions RQ and LQ were subtracted from the angles of the baseline condition. The differences were used for the statistical analysis.

### Statistical analysis

**Fig. 4:**
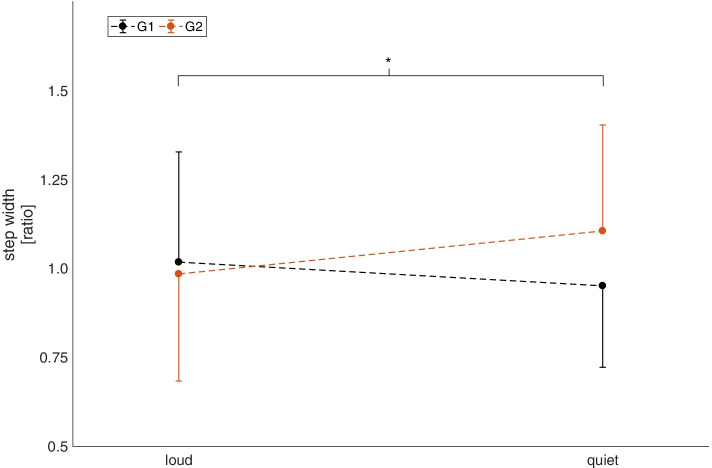
Step width of G1 and G2 at loud and quiet settings during the habituation period. Values are mean ± standard deviation. Significant interactions are marked with * (*p* < 0.05)

The results of the parameters are presented as mean values and standard deviations (mean ± SD). Only the deviation of the gait direction in Fig. 4 is given as mean values and standard error (mean + SE). A mixed ANOVA was applied to the temporal, spatial and directional parameters. The mental state (Bf-SR score) was analyzed using a sign test.

The data were checked by a Shapiro Wilk test for the condition of normal distribution. A Levene’s test was used to check for homogeneity of variances. The analyses were performed using SPSS (IBM SPSS Statistics, Version 26, Chicago, IL) and level of significance was set at α = 0.05. If a significant interaction effect was observed, post-hoc t-tests using Bonferroni correction were performed in MATLAB to identify detailed differences between conditions.

## Results

### Habituation period

Considering the *temporal parameters* of the habituation period, no significant effects were found for step duration, ground contact time, cadence, and stride speed (Table 1).

**Table 1:** Results of the habituation period

	loud		quiet		v	s	v*p	s*p	s*v	v*p*s
	left	right	left	right	*p*	*p*	*p*	*p*	*p*	*p*
**Stride length**										
G1	1.005 ± 0.035		1.013 ± 0.045		0.919	–	0.226	–	–	–
G2	0.996 ± 0.027		0.989 ± 0.040							
**Step length**										
G1	0.996 ± 0.049	1.006 ± 0.049	1.017 ± 0.074	1.007 ± 0.050	0.965	0.512	0.148	0.533	0.351	0.320
G2	0.991 ± 0.033	1.003 ± 0.043	0.980 ± 0.055	0.993 ± 0.052						
**Step width**										
G1	1.02 ± 0.310		0.98 ± 0.301		0.184	–	0.044*	–	–	–
G2	0.95 ± 0.229		1.11 ± 0.298							
**Gait direction**										
G1	−0.53° ± 3.60°		0.06° ± 3.29°		0.619	–	0.521	–	–	–
G2	0.36° ± 2.48°		0.30° ± 2.28°							
**Stride duration**										
G1	1.001 ± 0.050		1.001 ± 0.053		0.989	–	0.974	–	–	–
G2	1.999 ± 0.036		0.999 ± 0.039							
**Step duration**										
G1	0.992 ± 0.057	1.014 ± 0.052	1.001 ± 0.056	1.005 ± 0.048	0.572	0.880	0.596	0.269	0.535	0.660
G2	1.007 ± 0.041	0.991 ± 0.042	1.013 ± 0.039	0.994 ± 0.050						
**Ground contact time**										
G1	1.015 ± 0.053	0.999 ± 0.042	1.008 ± 0.039	1.005 ± 0.041	0.822	0.847	0.752	0.410	0.334	0.605
G2	0.992 ± 0.035	0.996 ± 0.032	0.994 ± 0.054	1.002 ± 0.032						
**Gait speed**										
G1	1.009 ± 0.082		1.018 ± 0.091		0.957	–	0.438	–	–	–
G2	0.996 ± 0.048		0.989 ± 0.066							
**Cadence**										
G1	1.006 ± 0.090		0.992 ± 0.085		0.531	–	0.399	–	–	–
G2	1.003 ± 0.028		1.005 ± 0.035							

Values are mean ± standard deviation. Gait direction is the difference of loud and quiet to the normal setting. All other parameters are loud and quiet relative to the normal setting. The *p*-values of the statistical analysis (ANOVA) are given in the right table section. The factors volume (v), side (s), the interaction volume*pitch (v*p), side*pitch (s*p), side*volume (s*v), and volume*pitch*side (v*p*s) were analyzed. The level of significance was set at α = 0.05. Significant differences are marked with a*

For the *spatial parameters*, no significant effects were found for stride length and step length. Regarding step width, no main effect of volume was found. However, an interaction effect of volume*pitch was found (F(1,30) = 4.39, *p* = 0.045, f = 0.38). This effect can be explained by a decrease in step width for G1 (high pitch) and an increase in step width for G2 (low pitch) from loud to quiet (Fig. 4). However, post hoc tests show no significant differences between the respective conditions.

### Asymmetry period

**Fig. 5:**
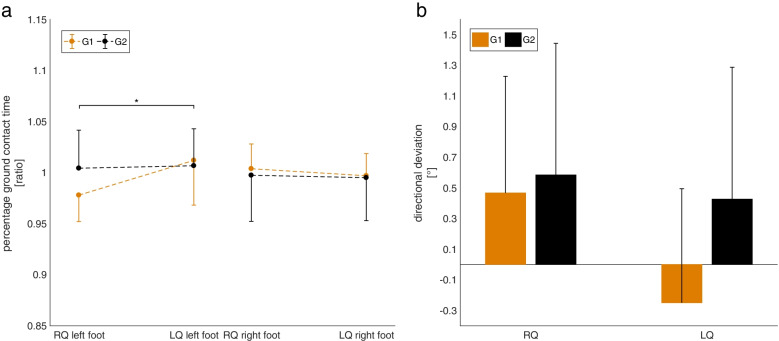
**a** Ground contact time of G1 and G2 at right quiet (RQ) and left quiet (LQ) settings during the asymmetry period. Values are mean ± standard deviation. Significant interactions are marked with * (*p* < 0.05). **b** Directional deviation of G1 and G2 at right quiet (RQ) and left quiet (LQ) settings during the asymmetry period. A positive value is defined as deviation to the left, a negative value as deviation to the right. No significant differences could be found. Values are mean + standard error

In the asymmetry period, there were no main effects of volume or side on the *temporal parameters* stride duration, step duration, ground contact time, stride speed, and cadence (Table 2). However, an interaction effect of volume*side (F(1,30) = 5.027, *p* = 0.033, f = 0.41) was found for ground contact time. Post hoc tests show a significantly higher ground contact time of the left leg of G1 (*p* = 0.046) for the LQ (1.004 ± 0.045) condition compared to RQ (0.978 ± 0.026). A similar trend can be seen for G2 (LQ: 1.004 ± 0.024, RQ: 0.997 ± 0.037), but here no significant difference can be found post hoc. The described effect is shown in Fig. 5 (left).

**Table 2:** Results of the asymmetry period

	RQ		LQ		v	s	v*p	s*p	s*v	v*p*s
	left	right	left	right	*p*	*p*	*p*	*p*	*p*	*p*
**Stride length**										
G1	1.000 ± 0.022		0.996 ± 0.026		0.768	–	0.883	–	–	–
G2	1.003 ± 0.019		1.002 ± 0.019							
**Step length**										
G1	1.002 ± 0.035	0.997 ± 0.033	0.992 ± 0.033	1.002 ± 0.041	0.970	0.226	0.435	0.287	0.806	0.178
G2	0.988 ± 0.047	1.016 ± 0.044	0.997 ± 0.037	1.016 ± 0.042						
**Step width**										
G1	1.048 ± 0.311		1.027 ± 0.281		0.314	–	0.641	–	–	–
G2	1.026 ± 0.350		0.963 ± 0.225							
**Gait direction**										
G1	0.47° ± 3.61°		−0.25 ± 3.44°		0.245	–	0.455	–	–	–
G2	0.58° ± 2.08°		0.43 ± 1.52°							
**Stride duration**										
G1	1.001 ± 0.020		1.002 ± 0.022		0.594	–	0.983	–	–	–
G2	1.002 ± 0.015		1.004 ± 0.013							
**Step duration**										
G1	1.008 ± 0.049	0.974 ± 0.064	0.990 ± 0.041	0.993 ± 0.050	0.400	0.930	0.357	0.239	0.318	0.286
G2	0.998 ± 0.038	1.014 ± 0.065	0.993 ± 0.025	1.006 ± 0.033						
**Ground contact time**										
G1	0.978 ± 0.026	1.012 ± 0.044	1.004 ± 0.045	1.007 ± 0.042	0.098	0.541	0.373	0.206	0.033*	0.084
G2	0.997 ± 0.037	0.995 ± 0.036	1.004 ± 0.024	0.997 ± 0.022						
**Gait speed**										
G1	0.993 ± 0.029		0.992 ± 0.038		0.617	–	0.747	–	–	–
G2	1.004 ± 0.034		0.999 ± 0.028							
**Cadence**										
G1	1.003 ± 0.049		1.005 ± 0.073		0.968	–	0.748	–	–	–
G2	1.002 ± 0.021		1.000 ± 0.013							

Values are mean ± standard deviation. Gait direction is the difference of right quiet (RQ) and left quiet (LQ) to the baseline setting. All other parameters are RQ and LQ relative to the baseline setting. The p-values of the statistical analysis (ANOVA) are given in the right table section. The factors volume (v), side (s), the interaction volume*pitch (v*p), side*pitch (s*p), side*volume (s*v), and volume*pitch*side (v*p*s) were analyzed. The level of significance was set at α = 0.05. Significant differences are marked with a*

For the *spatial parameters* stride length, step length, and step width, neither main nor interaction effects appeared in the asymmetry period.

Also, no significant main and interaction effects could be found for *gait direction*. Purely descriptively, however, a tendency of the study participants to walk in the direction to which the louder ground contact sound was heard can be detected (Fig. 5, right).

### Assessment of mental state

There was a significant decrease in the Bf-SR score (pre: 12.44 ± 7.28, post: 11.19 ± 7.29) from before measurements to after measurements (*p* = 0.045) indicating an improvement in *mental state*.

## Discussion

The present study intended to investigate the influence of the volume of real-time gait sonification on the gait pattern and gait direction of healthy young persons. The results show that an asymmetric volume of ground contact sonification directly influences the ground contact time unilaterally, which results in a temporal gait asymmetry. It can be seen that the ground contact time of the quiet foot is increased. However, no effects of the asymmetrical volume on spatial parameters of the gait, such as step length, and walking direction when walking blindfolded were found. Considering the overall volume during the habituation phase an effect on the step width was revealed, which seems to interact with the pitch of the gait sonification: for G1 (high pitch) a positive relationship between volume and step width, but for G2 (low pitch) a negative relationship becomes apparent. In addition, the Bf-SR survey showed that the mental state of the study participants improved from the beginning to the end of the measurements. It is clear that this development is not due to the sound of the sonification, as no differences between the groups can be detected in this development. Presumably, the improvement in mood is rather due to the task itself or to its accomplishment.

Previous studies on volume indicated an influence of this parameter on spatio-temporal perception [2, 61–65] and, to a limited extent, on human kinematics [66]. However, we are currently not aware of any studies investigating the influence of volume in gait sonification. In order to make a first step towards a better general understanding of the influence of volume on the effectiveness of MS, explorative hypotheses were tested.

In a first consideration of the results, it seems surprising that volume modification in the asymmetry period did not affect spatial parameters, although volume is predominantly associated with spatial distances, directions, and velocities [64, 65, 75]. The reason why the volume affected the gait pattern of the participants only in the habituation period might be due to a high degree of automation of the gait, which prevented an adjustment to a possibly less economical gait pattern. Also, the unilateral modification of the auditory stimulus in the asymmetry period might have been too small to affect spatial parameters and/or might have been overlaid by proprioceptive, tactile, and vestibular afferences.

We tried to make the volume difference between the two sides as large as possible but still not noticeable to avoid participants’ intentional motion adaptation. Only three of the 32 participants reported having detected a volume difference after the measurements. Several questions follow in this regard. First, whether knowledge of or recognition of asymmetric volume interferes with (unconscious) motor adaptation. And, if this is the case, to what extent verbal instruction (e.g., “Do not consciously adjust your movement to the sonification.”) could counteract this. Second, the question of optimal volume difference arises. It is possible that the effect on ground contact time that occurred correlates with the volume difference, similar to reaction time tasks in which lower reaction times can be observed with louder acoustic stimuli [76–78], although here a comparison regarding the application of the sound and the motor response is not obvious. If an analysis of the effect size of increasing volume difference on gait symmetry is successful, this correlation could be a crucial factor in making the use of gait sonification efficient in rehabilitation. However, it should be noted in this context that elderly patients in particular, who could benefit from gait sonification e.g. after stroke, Parkinson’s disease, or arthroplasty, often suffer from hearing loss. If this hearing loss is more pronounced on one side, the volume difference must be adjusted accordingly or even overcompensated to compensate for habituation effects. Finally, based on the results presented here, it can be assumed that the gait pattern of patients with unilateral hearing loss might suffer from the hearing impairment. Although no studies are currently known on laterality, preliminary evidence suggests that hearing impairment leads to increased risk of falls in the elderly [79, 80]. Again, the use of gait sonification with volume settings adapted to the user could potentially counteract deterioration of gait due to hearing impairment.

With regard to the impact of volume on step width, which occurs contrarily for the two different pitches, the influence of pitch on movement and a possible interaction between pitch and volume should also be considered. In an early work by Wood [81] it became evident that in human perception there is an interaction between pitch and volume that can affect movement reactions. In the experiment, reaction times were measured after hearing a simple syllable that varied in pitch and volume. One-dimensional changes in pitch and volume showed shorter reaction times than orthogonal-dimensional changes in pitch and volume. Similar psychophysical correlations between pitch and volume could also be found for non-speech-related sounds [82, 83]. This interdependency of pitch and volume might be an explanation for the divergent step width change at low vs. high pitch and increased volume.

Gomez-Andres et al. [50] also showed that the overall pitch of acoustic gait feedback influences the gait symmetry of stroke patients. Here, a high pitch of amplified footsteps sounds increased the asymmetry of the patients’ ground contact times, while a low pitch reduced the asymmetry. Although a different method of sound generation respectively amplification and other participants were chosen in Gomez-Andres et al., the current results show similarities regarding the effect of different pitches on gait symmetry.

Furthermore, in the present study, the results of the asymmetry period show a clear effect on the temporal parameter ground contact time. Since only the ground contact time was presented acoustically, it can be assumed that the sound-motion relationship was clearly recognizable to the participants and that sonification had a direct influence on gait pattern. The mechanism underlying this influence of gait sonification has been investigated and discussed in previous studies. It is hypothesized that the mapping of sound to movement leads to audio-motor coactivation in the CNS. This coactivation occurs because the acoustic stimuli are directly generated by the user’s movement, probably unconsciously [5, 30, 84]. Due to this close audio-motor coupling, it is possible that continuous sensorimotor adaptation takes place and, as explained by the forward model, movement adaptation occurs [50]. Regarding the observed effect on ground contact time, it should additionally be considered that the human auditory system perceives rhythmic information and temporal structures particularly clearly [85–87], which might have led to a stronger effect on motor timing compared to range and direction of motion. Thus, the temporal increase in ground contact time might have been favored with reduced volume.

In the present study, it can also be assumed that a comparison of the actually perceived sensory information (afferent input) with the expected sensory information (efference copy) led to a discrepancy. An attempt was made to compensate for this by changing the ground contact time. Since the participants were not informed about the volume modification, it can be assumed that the processes described were mainly unconscious. The forward model could therefore explain the observed effects in the case of a repetitive and automated movement such as the human gait. Especially since in the present study visual information was reduced during walking and subjects relied heavily on sonification as auditory information to maintain automated processes [88].

It must be regarded as a limitation of this study that it cannot be assessed whether the ground contact time was a result of altered ground reaction forces due to the lack of force/pressure measurement. Possibly a stronger heel strike or a more intensive push off led to an extension of the ground contact time during the quiet sound condition. The participants (unconsciously) could have tried to produce a louder sound by applying more force. An additional use of force or pressure plates should clarify this question in the future. Furthermore, it might be useful to replicate the results using a larger sample. This could also clarify whether there might be a statistically significant effect of volume on gait direction when walking blindfolded.

## Conclusions

The present study showed that the volume of gait sonification has directly affected the gait pattern of healthy young persons. At asymmetrical volume, a unilateral increase in ground contact time was observed on the side with reduced volume. Also, an interaction of pitch and volume was observed mainly with an overall change in volume. This could be explained in terms of psychophysical perception, which should be considered when using volume for gait sonification. We thus provide first clues for an appropriate sound-motion mapping and a targeted use of volume. Based on the present results, we would recommend for gait sonification that temporally asymmetric parameters be presented directly acoustically on both sides and that the side on which the movement is performed in a shortened manner be presented more quietly than the other. In this way, the user would respond by amplifying the movement, i.e., increasing its duration, which would improve temporal movement symmetry. A lasting effect of volume modification must be investigated in future intervention studies. In this context different patient groups should be considered. The available findings can be helpful to improve the effect of gait sonification in patients with asymmetrical gait pattern and thus to return to a physiological gait more quickly and easily.

## Data Availability

The datasets during and/or analyzed during the current study available from the corresponding author on reasonable request.

## Abbreviations

G1: Group 1 (150-250 Hz)
G2: Group 2 (95-112 Hz)
RQ: Right quiet
LQ: Left quiet
TD: Touch down
TO: Toe off
MS: Movement Sonification
IMU: Inertial measurement unit
Bf-SR: Mental state scale [74]
